# “Exceptionally challenging time for all of us”: Qualitative study of the COVID-19 experiences of partners of diplomatic personnel

**DOI:** 10.1371/journal.pone.0293557

**Published:** 2023-11-02

**Authors:** Samantha K. Brooks, Dipti Patel, Neil Greenberg

**Affiliations:** 1 Department of Psychological Medicine, King’s College London, London, United Kingdom; 2 Overseas Health and Welfare, Foreign, Commonwealth and Development Office, London, United Kingdom; Western University Faculty of Science, CANADA

## Abstract

**Background:**

Although the romantic partners of diplomatic personnel frequently accompany their spouses to overseas postings and face the challenges of having to adjust to new cultures and separation from friends and family, they have rarely been the focus of academic research. This study explores the lived experiences of the partners/spouses of diplomatic personnel from the United Kingdom’s Foreign, Commonwealth and Development Office (FCDO) during the COVID-19 pandemic.

**Methods:**

Partners of FCDO staff took part in semi-structured interviews about how COVID-19 had affected their lives and their perceptions of the organisation’s response to the pandemic. Thematic analysis was used to analyse the data.

**Results:**

Eleven partners of FCDO staff took part, who between them had lived in 14 different countries during the pandemic. The analysis identified six key themes: deployment-specific challenges such as travel restrictions, quarantine and evacuation; children; impacts of the pandemic including financial and psychological; perceptions of the organisational response to COVID-19; support and help-seeking; and suggestions for the future. Overall participants reported experiencing a number of challenges, many of which left them feeling powerless and not in control of their own lives. Participants frequently described a lack of clarity around policies and support. Social support appeared to be valuable, but many participants wanted more support from the organisation and from informal networks.

**Conclusions:**

Diplomatic (and similar) organisations could enhance the wellbeing of the partners of their staff through improved communication and support. Keeping families informed about restrictions, requirements, policies and available help during a crisis, and reaching out to them to offer advice and support, would likely be beneficial. It is important that lessons are learned from the COVID-19 crisis in order for organisations to be able to support their employees and families if another prolonged crisis were to occur.

## Introduction

The romantic partners of professionals whose roles involve regular international travel frequently accompany them on overseas postings, often disrupting their own lives, careers and social networks [1]. Many partners experience uncertainty, change (e.g., in their working conditions, financial status, and social activities), feelings of a loss of control, and isolation from familiar support networks [2, 3] all of which can have a detrimental effect on mental health and wellbeing.

The partners of diplomatic personnel are one such group who frequently accompany their spouses on overseas postings. Diplomats’ romantic partners are a unique group as the overseas postings they accompany their spouses on tend to last only a few years at a time. Over the course of a diplomatic career, relocations are relatively frequent and repeated, with families often having to move to new posts in new countries just as they have settled into their previous posting environment [4]. Relocation has been recognised as a particularly stressful event for spouses because it is a major life event involving a simultaneous reduction in coping resources (such as social networks) and potentially negative alterations in sense of identity [5]. Such frequent relocations can make it difficult to establish a stable life, plan for the future or establish a career. Additionally, repeatedly having to adapt to new cultures and new social norms (and potentially needing to learn new languages) can be emotionally draining [6]. Spouses, particularly those without a strong support network, can struggle to adjust [4]. Compared to other expatriate spouses, the partners of diplomatic personnel are likely to have unique experiences specific to diplomatic life, such as the need to manage political sensitivities and to be seen as a representative of their home country, and may be affected by political tensions or security concerns [7]. Additionally, diplomatic spouses may feel they have a greater responsibility to engage in community relations in their host countries than other expatriate spouses, due to the importance placed on spouses in the social aspects of diplomatic life [7].

There is very little published literature on diplomatic spouses, but one study [4] found that few diplomatic spouses reported receiving cross-cultural training and subsequently many found it difficult to adjust to new cultures. While diplomatic spouses have rarely been the focus of academic research, there is a wealth of literature on military spouses who are likely to have somewhat similar experiences to the partners of diplomats, in that those accompanying their partners experience frequent, repeated relocations, leaving behind family and friends and needing to adjust to new cultures and customs. Much like diplomatic spouses, military spouses are frequently expected to fulfil social engagement roles, act as an unpaid assistant to the service member, take on unpaid roles performing emotional labour in the community, and provide stability at home by performing domestic and caring tasks [8, 9]. Research suggests military spouses frequently experience worry, loneliness, adjustment difficulties and feelings of a loss of control over their lives [10–12]; they often find it difficult to establish support networks or form long-term friendships given how frequently they move around [12], and those who do not manage to build connections are at risk of isolation, psychological distress and mental health problems [13]. Many give up their careers, struggling to find employment or having to take lower-level jobs [12] which can create a sense of loss of status [14, 15].

These experiences may negatively affect the mental health and wellbeing of spouses, although it may be that the potentially social supportive overseas diplomatic environment could be protective. Gudmundsdottir et al. [4] found that partners of diplomats with strong support networks had better adjustment and satisfaction with life, supporting previous research suggesting social support is important for spouses’ adjustment [14, 16–18]. If spouses are struggling to adjust, this can also negatively impact on the employed partners’ productivity and performance at work and can lead to early return from assignments [19]; therefore, family support issues are recognised as crucial to the success of the assignments of the employed partners [20].

Since 2020, expatriate families have had an additional challenge to cope with: the outbreak of novel coronavirus SARS-CoV-2 (COVID-19) was declared a global pandemic on March 11^th^ 2020 [21] and became an unprecedented global crisis which saw almost every country in the world ‘lock down’ with restrictions on mobility and social contact. During the pandemic, many partners of diplomats will have found themselves ‘stuck’ at an overseas posting away from their friends and extended families, separated from their partners due to evacuation, and/or taking on extra caring responsibilities such as childcare or home-schooling.

To date, relatively little is known about the COVID-19 experiences of partners of international travellers. To our knowledge, there is no published academic literature exploring the impact of COVID-19 on diplomatic spouses; again, we can turn to military literature given the potential similarities between diplomatic spouses and those of Armed Forces personnel. Research suggests the pandemic has intensified many of the pre-existing concerns common within military families–such as military spouse unemployment/under-employment, time away from families, child-care challenges and financial concerns [22].

Emerging literature which does examine the impact of the pandemic on the spouses themselves shows evidence of military spouses experiencing greater depression, generalised anxiety disorder, alcohol use disorder and post-traumatic stress disorder than the general public [23]. Poor mental health of partners of military personnel during the COVID-19 pandemic has been found to be predicted by household financial impact, changes in work situation, having children under the age of 18 in the house and children experiencing emotional, behavioural or other difficulties [24]. However, there are also indications of the resilience of military families: Fanari et al. [25] found that, while the uncertainty of the pandemic was described as stressful by military spouses, many felt that they had a pre-existing community unique to military spouses and so were well-prepared for drawing on that community during times of crisis. Overall, little research has been published so far examining the impact of COVID-19 on expatriate spouses, and there is notably a particular lack of qualitative research allowing for a deeper understanding of aspects of the pandemic which are particularly stressful and the impact they have on spouses’ psychological wellbeing.

Studies on other, non-diplomatic sojourners have identified a number of stressors and coping strategies associated with the pandemic. Many of these are also likely to have affected diplomatic spouses. For example, college students repatriated from ‘study abroad’ experiences identified numerous stressors such as the financial costs associated with repatriation, isolation during quarantine upon re-entry to their home countries and uncertainty around what the future might hold [26]. These stressors led to feelings of distress, loneliness, and confusion [26] and, where individuals had a particularly difficult time readjusting, predictive of longer-term mental ill-health [27]. This provides an idea of the difficulties that evacuated diplomatic spouses may have experienced and how they may have been affected. Fanari and Segrin [26] also found that keeping busy, seeking social support and reframing the pandemic experience in a more positive way were key coping strategies.

The goal of this study was to explore the COVID-19 experiences of diplomatic spouses. Since the pandemic was a prolonged crisis, it is important to consider existing models of cross-cultural adjustments and transitions and how they relate to periods of crisis. Notably, Kim’s integrative communication theory of cross-cultural adaptation [28] describes a process model of how expatriates’ adjustment to their new countries develops over time. The model suggests that stress is experienced when entering a new country due to tension between seeking congruence with the new culture and wanting to hold on to aspects of home culture. This stress then drives individuals to adapt and grow; given that new challenges are always arising, there is a constant cyclic process of stress, adaptation, and growth. According to Kim’s theory [28], each new stressful experience involves a temporary setback which activates energy to re-engage and adapt. The COVID-19 pandemic is likely to have been a particularly severe, prolonged stressor, different to any which had been experienced before. Investigation is therefore needed to establish the impacts of such an extreme stressor and the unique coping strategies which may have been drawn upon to allow individuals to adapt. According to resilience literature, the capacity to adapt and cope with adversity is important in allowing individuals to either ‘bounce back’ to their pre-adversity state or to adapt to their new situation in a healthy way [29]. Therefore, understanding the needs and experiences of diplomatic partners during the COVID-19 pandemic helps develop understanding of how they can best be supported and how resilience can be fostered in this particular, unique group.

The current research qualitatively explores the experiences of the partners of diplomatic personnel during the COVID-19 pandemic. Using semi-structured interviews with partners of employees of the United Kingdom’s Foreign, Commonwealth and Development Office (FCDO), we investigate how the wellbeing of diplomatic partners was affected by the pandemic and their perceptions of the organisational response to the pandemic. The overall aim of this study was to use these findings to develop recommendations for how the FCDO–and similar organisations–could best support their staff during a prolonged crisis such as a pandemic.

## Method

### Design

This study collected qualitative data using semi-structured interviews, which contained central, open-ended questions to be asked to each participant while also allowing participants to elaborate as they wished and direct the flow of the interview [30].

### Participants

Eligible people were aged 18 or over and had a partner employed by the Foreign, Commonwealth and Development Office (FCDO) both currently and for at least six months. In order to recruit participants with a broad range of experiences, there were no inclusion criteria relating to whether participants had been abroad or in the United Kingdom (UK) during the pandemic, the countries they were based in, or the grades/roles of their partners within the FCDO. The study was deliberately designed with broad inclusion criteria in order to gain insights into the experiences of as wide a variety of participants as possible, including those who remained at post, those who were evacuated back to the UK, and those who spent at least some of the pandemic in local posts.

In total, twenty-one partners of FCDO staff contacted the researchers for further information about the study. As we do not know how many partners saw the DFSA advertisement or how many FCDO staff shared the invites with partners, it is not possible to calculate an overall response rate. Of the 21 who contacted the researchers, eleven (52%) took part. Five (45.4%) were male and six (54.5%) were female; ages ranged from early 20s to mid-60s (mean 45) and their partners worked in a wide range of roles and grades within the FCDO (no specific details are presented in order to protect the identities of participants). Between them, participants had resided in 14 different countries across six continents during the pandemic (this number is greater than the number of participants, as several had relocated at least once during the pandemic). Four participants had spent at least some of the pandemic in the UK; other regions participants had lived in included Asia (n = 4), Africa (n = 3), Europe (n = 2), Oceania (n = 2) and North America (n = 2). Six (54.5%) reported having deployed to ‘hardship locations’ (locations with extremely difficult living conditions where they received hardship allowance). Details of specific countries are not presented in order to protect the anonymity of the participants.

Interviews lasted between 22 minutes and 52 minutes (median: 37.5 minutes), providing an overall data corpus consisting of almost seven hours of interview talk.

### Recruitment

An invitation letter was created by the authors which summarised the aims and proposed methodology of the study and provided the researchers’ contact details, asking anyone interested in taking part to contact the researchers of their own volition. As we were recruiting FCDO staff themselves for a similar study at the same time, the invitation letters explained that we were interested in recruiting both staff and their partners and invited any staff who received the invite to share it with their partners. In September 2021, welfare staff at the FCDO emailed the invitation to 100 randomly selected diplomatic staff who met the inclusion criteria and due to low response rate, two further rounds of invitations were sent in November 2021 and January 2022. The Diplomatic Service Families Association (DSFA) newsletter also published the invitation during this time period. Full Study Information Sheets and Consent Forms were sent to all individuals who contacted the researchers volunteering to take part or asking for further information, and interviews were arranged via email. Recruitment ended when data saturation was considered to have been achieved, i.e., no new information was emerging from interviews.

### Interviews

The authors developed an interview guide of key questions to be asked. As this was part of a larger study on diplomats’ and partners’ experiences, there were a wide range of topics covered in these questions. Those relevant to this paper included questions about the impact of COVID-19 and perceptions of support available. For example, questions included *‘Can you tell me if*, *and how*, *your day-to-day life has changed as a result of COVID-19*?*’* and *‘In respect of seeking help through the FCDO*, *what support options do you know might be available for partners*?*’*. All participants were also asked at the end of their interviews whether they had any additional points or comments to make which had not already been covered. Interviews were carried out by the first author between September 2021-February 2022. Most (n = 10) took place over Microsoft Teams whilst one took place over Zoom due to the participant lacking access to Teams. It was felt that good rapport was built between interviewer and interviewee, beginning at the initial recruitment stage which friendly emails to arrange interview times. At the start of the interviews, rapport was built further while the interviewer checked participants had understood the Information Sheet, confirmed the purpose of the interview, thanked the participants for agreeing to take part and reassured them of confidentiality. The semi-structured nature of the interviews also facilitated rapport, allowing the interviewer to take time listening to what each participant felt was particularly important to them, allowing them to shape the flow of the interview, and responding to participants with empathy and respect. All interviews were recorded and transcribed verbatim by the first author with identifying information removed from the transcripts and replaced with ‘[redacted]’.

### Ethics

The study adhered to British Psychological Society (2018) guidelines [31]. Prior to the interviews, all participants received full Information Sheets and provided written informed consent to take part. Their initial invitations made it clear that participation was voluntary and there would be no consequences for declining to take part. Participants were reassured they could stop the interviews if they wished or withdraw their data from the study up to a month after the interviews. They were also reassured that no identifying details would be made public in any subsequent publications and that the FCDO would not, at any point of the study, know who had taken part. Only the first author had access to identifying information at any point during the study, and as soon as interviews were transcribed, they were anonymised. All data was processed in accordance with the General Data Protection Regulation 2016 [32]. The research was approved by the Psychiatry, Nursing and Midwifery Research Ethics Subcommittee at King’s College London (ethical clearance reference number: HR/DP-20/21-22511).

### Analysis

Transcripts were imported to NVivo software (Version 12; QSR International Pty Ltd, 2018) [33] where data were analysed by the first author according to the principles of inductive thematic analysis [34]. This approach involved the six stages described by Braun and Clark [34]. First, multiple readings of the transcripts took place in order to familiarise the author with the data (Stage 1: Familiarisation). Next, initial codes were generated by breaking the transcripts down into small chunks of data based on their content (Stage 2: Generating initial codes). These initial codes were then examined in detail and collated into overarching themes–basically, considering how different codes may combine into an overarching theme (Stage 3: Searching for themes). Stage 4 (Reviewing themes) then involved a deeper review of themes to ensure they reflected the data corpus, and also that themes were coherent with each other while also distinctive from each other. Stage 5 (Defining and naming themes) involved the first author giving each theme a definition and name to appropriately capture their content; these were discussed with the wider research team. The final stage (Producing the report) entailed choosing quotes to appropriately illustrate the themes in the write-up. Using NVivo, we were able to view all quotes within each theme together, and read these multiple times. Being able to view all quotes together made it easier for us to choose quotes which illustrated the themes well and captured what we believed to be the general feelings of the participants from our impression of the interviews. We deliberately chose quotes which we felt were not so short that it raised questions about what they truly illustrated, but not too long that they overwhelmed the manuscript. We also deliberately chose quotes that were articulated well and would be easily understood by readers without requiring too much clarification by the authors. Finally, we also ensured that each individual participant had at least one direct quote presented in the manuscript, to ensure each person’s voice could be heard.

Trustworthiness of the thematic analysis was enhanced by ensuring all authors agreed that the themes reflected the data; presentation of direct quotes to support analysis; and documentation of the analytic process through an ‘audit trail’ of raw data, transcripts and reflective memos [35].

### Data saturation

As stated, we ended recruitment when ‘data saturation’ was believed to have occurred. Literature suggests there are a number of factors facilitating data saturation [36]. Firstly, a simple research question is more likely to achieve saturation quickly than a more complex question [36]. Given the paucity of literature on diplomats’ spouses in general and especially during the pandemic, our research question was simple and broad, with the aim of understanding the general pandemic experiences of this population. Secondly, the researchers’ experience of qualitative methods can influence how quickly saturation is likely to occur, with more skilled researchers more likely to have the knowledge and experience of how to construct their interview questions in order to obtain the data they need [36]. The first author, who was responsible for the data collection and coding, has a wealth of experience over the last two decades of conducting qualitative research and thus was considered to have the expertise necessary to obtain the necessary data quickly and efficiently. Based on these two factors, we did not expect to require a very large study population. Nevertheless, we made efforts to ensure that the decision regarding when data was considered ‘saturated’ was as objective as possible. The first author noted their initial thoughts about potential codes and themes immediately after each interview, in NVivo memos. Transcription and full coding were carried out after every three interviews–so for example, when we carried out the fourth interview, we were already aware of the initial codes identified in the previous three interviews. After the seventh, eighth and ninth interviews, the memos suggested that while new data was allowing us to elaborate on previously identified codes, it was not providing *new* codes. After full coding of nine interviews, we confirmed that new codes were not being identified from the data. Rather, different aspects of existing codes were emerging based on participants’ unique situations–for example, we named one code ‘travel challenges’, and we noted that the specific circumstances and impacts of these challenges might differ depending which country participants were based in, but overall there were no *new* codes relating to travel. In other words, we found recurring codes, with greater richness–but not entirely new codes–emerging as we expanded the data corpus. Accepting that it would not be possible to capture every possible experience of diplomatic spouses as we did not have the time or resources to interview participants from every country in the world, we made the decision that ‘no new codes’ was indicative of data saturation. We believed that no new codes were emerging after nine interviews, but given that we had additional willing participants who had shown interest in the study, we carried out two additional interviews to confirm this.

### Reflexivity

Immediately after each interview, the interviewer (first author) created detailed reflective memos in NVivo to record observations of the interviews: these included the most salient issues arising from the interview, initial thoughts about potential themes arising from the data, and overall thoughts on the interview. These were shared with the wider research team allowing them to reflect on the interview technique; consider whether any questions should be added, removed or improved; and reflect on how their own experiences or expectations may have influenced either their interactions with participants or their interpretation of the data. We acknowledge that the interviewer may have had assumptions about what the results might show, prior to the study, based on what is known from the extant literature and their own experiences of conducting research with military, law enforcement, and other governmental departments. However, throughout the interviews they consciously questioned these assumptions and encouraged participants to talk freely about their own experiences and perceptions. Additionally, prior to data collection the interviewer had very little specific knowledge or understanding of the FCDO, its policies or organisational culture–being neither a diplomat nor diplomatic spouse themselves. This meant they had no assumptions about what diplomatic life entailed or how the organisation would be perceived by participants. While the interviewer obviously had the experience of COVID-19 in common with participants–a shared experience which further bolstered the rapport developed between interviewer and interviewee–they had no prior understanding of how the pandemic might have affected individuals in the unique position of having to frequently relocate. Overall, the first author was chosen to be the interviewer above other members of the research team due to their lack of experience, and thus lack of assumptions, about diplomatic life–meaning that the findings could be interpreted as objectively as possible.

## Results

A summary of the themes and sub-themes identified through thematic analysis is presented in Table 1.

**Table 1:** Themes and sub-themes.

Theme	Sub-theme
Deployment-specific challenges	Travel requirements and restrictions; Challenges of quarantine; Experiences and impacts of evacuation
Managing disruptions and difficulties associated with the caregiving of children	Childcare and schooling; Difficulties regarding older children; Children’s health
Impacts of the pandemic	Financial impact; Psychological impact; Living in lockdown
Perceptions of organisational response to COVID-19	Perceptions of organisational communication; Provision of vaccines; Availability and perceptions of organisational support; Barriers to organisational help-seeking
Informal support and help-seeking	Diplomatic Service Families Association; Other informal support networks
Suggestions for the future	Learning lessons from the pandemic; Understanding the importance of flexibility

For the purposes of writing up the analysis, participants were each given a unique identification code (S1 –S11) to protect their identities. An ellipsis in brackets within a quote indicates that some ‘filler text’ has been removed in order to shorten the quote without changing the meaning of what was said. Text in square brackets within a quote indicates text which has been inserted by the authors for clarification.

### Deployment-specific challenges

#### Travel requirements and restrictions

Unsurprisingly, participants reported experiencing numerous travel-related challenges during the pandemic. The many requirements for travelling were perceived as stressful and left participants feeling powerless: *“All the paperwork that’s needed to get out to post (…) the stresses of having a COVID test and then making sure (…) the results will be back within seventy-two hours or forty-eight hours of travel and all of these things begin to sort of weigh you down a little bit (…) you’re completely out of control*, *you cannot control any of these factors” (S8)*. Travel restrictions meant diplomatic families had to understand and comply with the different rules and requirements of each country they transited which was reported to be stressful; indirect flights were perceived to be particularly difficult when travelling with young children or with lots of luggage: *“carrying all your belongings with you on a direct route is fine but when you are disrupted due to the pandemic you cannot be agile with all of your baggage with you” (S10)*. Overall, the many restrictions in place for travel left participants reluctant to travel at all which resulted in feeling isolated and stuck: *“There is a sense of isolation that a lot of people have felt*, *you cannot go back to the UK easily*. *There are so many loopholes and so much paperwork that you have to have in hand to get back (…) it actually puts people off going back to the UK” (S8)*.

Attempting to fly into new postings in COVID-free countries was particularly stressful due to the extremely limited flights into these countries, with only flights from other countries which had been COVID-free for two weeks allowed. Thus, anyone trying to fly to these countries had to constantly ‘shield’ and live in fear of catching COVID-19, sometimes for months on end due to late cancellation of flights. One participant described how they and their partner had spent months moving around various countries in order to be able to fly into a COVID-free country, which they described as *“basically trying to outrun a virus for the past six months” (S10)* which was *“detrimental to our mental health” (S10)*. They also described having to self-isolate every time they needed to fly, explaining how *“you’re constantly in quarantine because it’s like if (…) we get a positive test we can’t fly (…) [we are] living like as if COVID is everywhere” (S10)*. This caused stress and not feeling in control of their life: *“It’s really hard for people to understand what that negative test is doing to us*, *we couldn’t leave the house (…) that was the most stressed I think I’ve ever been because you can’t stop it*, *you can’t make the test say negative if you have COVID (…) you’re completely out of control of that situation” (S10)*.

Many expressed frustration at the apparent disconnect between many countries’ governments making the decision to ‘live with COVID-19’ and relax many restrictions, and yet also still having extremely strict rules around travelling, meaning that people who travel frequently are not really ‘living with COVID-19’ at all. For example, one participant explained *“if we’re going to live with COVID*, *then we have to be able to fly with COVID or go home with COVID or go to work with COVID (…) either we’re living with it or we’re not living with it*. *You can’t say we’re living with it but our whole life is ruined because everyone has COVID around us and we can’t get a flight (…) we are not ‘living with COVID’*, *we are living in fear of testing positive*. *We have managed to never catch it*, *but at what cost to our mental health*?*” (S10)*. Many perceived that the FCDO could have done more to arrange diplomatic exemptions for travel and quarantine: *“My personal view is that the organisation didn’t negotiate an exemption when legislation was going through about travel internationally and I don’t know why” (S4)*. Several participants thus felt they were treated no differently to tourists, despite needing to travel for their partners’ work, and they described feeling that diplomats (and their families) were *“lumped in the same bracket as normal tourists arriving back from holiday” (S8)*.

#### Challenges of quarantine

Needing to quarantine on arrival to new countries was described as stressful, tedious, and lonely, with several participants describing strict hotel quarantine rules such as *“Brits weren’t even allowed out after day five when you had your second PCR test in the hotel for thirty minutes exercise*. *So literally you’re stuck in a hotel room not able to open the windows*. *The only time you can open the door is two to three times a day to pick up the food that’s left on a tray outside” (S8)*. This was described as having a detrimental effect on wellbeing leaving participants feeling not in control of their lives: *“there’s sort of total sense of complete lack of control*. *There’s nothing you can do about it other than put up with it (…) this sense of having no control over it (…) when you’re in quarantine*, *little issues suddenly become magnified” (S8)*.

Most described frustration with hotel quarantine requirements even if they had accommodation waiting for them and explained that they did not understand the rationale for this, perceiving that they would be safer in their own houses than in hotels. For example, one participant described how on arrival at their partner’s posting they would have to quarantine for *“three weeks in a hotel room without leaving it” (S10)* despite the fact that *“we have (…) a house [at post] waiting for us*, *it’s a detached house*, *it has a security guard in the front twenty-four seven*, *like surely we could stay in that house*, *the two of us (…) I just feel like being in a hotel is actually more risk than being in a detached house” (S10)*.

Needing to quarantine when travelling for compassionate reasons, or other non-tourism reasons, was described as particularly psychologically challenging as participants missed out on spending time with family members: *“[Partner’s parent] was diagnosed with terminal cancer so we had to go back (…) to the UK*, *isolate for ten days which ate into the time we were hoping to spend with [partner’s parent] (…) that’s obviously had a bit of psychological impact on us” (S8)*.

#### Experiences and impacts of evacuation

One participant described having been evacuated–without their partner–from an overseas post back to the UK, which was described as a particularly stressful and upsetting situation: *“the Foreign Office basically forced*, *well (…) did force the kids and I to leave [Southern African country] (…) at the end of March 2020 (…) which was obviously pretty traumatic for the kids and I and my wife (…) exceptionally challenging time for all of us” (S1)*. Being forced to return to the UK without their partner led to the feeling they had no agency over their own life: *“I’m an adult*. *I feel I’m capable of making my own decisions about my life and to be told by a lawyer in London that actually no*, *you don’t have agency (…) baffling” (S1)*. Being evacuated was also viewed as being hugely detrimental to the partners’ own employment: “*the Foreign Office forcing me to leave meant that I had to abandon all of my work (…) which meant that my clients were unhappy and so we lost contracts and we lost opportunities for more contracts (…) I’ve sort of hesitated to even calculate how much it cost our business” (S1)*.

Being evacuated from countries with relatively few COVID-19 cases, back to the UK which had far more cases of infection was frustrating: *“when they forced us to leave there were two cases of COVID in [Southern African country] confirmed and there were about forty thousand (…) in the UK (…) why are you forcing us into a position of risk*? *(…) you’re increasing our chances of getting COVID exponentially” (S1)*.

The decision to evacuate partners and children was described as having a substantial, potentially long-term impact on families, causing them to reconsider what they would do in the future: *“I think that (…) has left a mark on us as a family but also (…) made us think differently about what we want to be doing next*, *and (…) how that’s going to work” (S1)*.

### Managing disruptions and difficulties associated with the caregiving of children

#### Childcare and schooling

Most participants described their partners’ workloads increasing during the pandemic. This meant that they had to sacrifice their own time to ensure their families were still cared for: *“The same amount of work was flying around the system*, *irrespective of the fact that people had to look after their five-year-olds (…) it relies very heavily on the partners taking a massive massive heavy lift” (S4)*.

One participant who had accompanied their partner to a hardship posting reported being unable to fly their children out to post from their boarding school in the UK *“because we were unable to provide good enough Wi-Fi or electricity for them to come back to post and do their education here” (S3)*. This created many additional problems for the family on top of the strain of being separated. For example, the participant needed to fly back to the UK during every school holiday in order to take care of their children which meant that they needed negative COVID-19 tests in order to be able to fly back to the UK, meaning they felt forced to self-isolate frequently to avoid catching the virus. As a consequence, they had to give up their work during the pandemic because it required face-to-face meetings with clients; *“I’m not prepared to go into other people’s houses during the pandemic because we’ve got kids in boarding school in the UK*. *I have to travel backwards and forwards all the time*. *So I’m basically living in semi-isolation because I cannot afford to get [COVID] because then I can’t fly and I can’t look after my children during the holidays” (S3)*. The frequent travelling back to the UK to look after the children was also reported to have had a substantial financial toll: *“I’ve had to rent a property*, *I haven’t had good enough Internet (…) to be able to contact people and do FaceTime calls ’cause I’ve been in holiday accommodation*. *I’ve rented cars (…) in the end I bought a car because I was going backwards and forwards so much (…) some families have spent tens of thousands of pounds because their children have not been able to come out to post and therefore the parents have had to go back every time*, *rent properties and isolate” (S3)*.

Some participants also described the challenges of home-schooling: *“A lot of spouses who are coping with the home-schooling have found it very difficult because there’s been no end*, *I mean fifteen months is a long time to be home-schooling your children (…) I know a lot of spouses have had stresses and strains*, *some of whom have got their own jobs to do (…) for some parents it has actually been very difficult to juggle work commitments and home-schooling” (S8)*.

#### Difficulties regarding older children

One participant with a child at university in the UK reported not being able to fly their child over to be with them during the pandemic due to their child being over eighteen (and so not classed as a dependant) and not being a resident of the country the participant was based in: *“We were told by the Foreign Office our daughter wouldn’t be entitled to residence because she (…) doesn’t live here full-time (…) we couldn’t get her back (…) she’s not got any (…) house in the UK*, *she’s not got any income (…) it was a really distressing time (…) she was very much still a dependant (…) adult only in the word ‘adult’ you know” (S11)*. This situation, where the participant’s child was not recognised as a dependent by the FCDO due to being over eighteen, had caused immense stress to the participant; *“I had a weekend of just crying” (S11)*. They felt that the organisation should expand their definition of dependants to children still in full-time education, rather than simply ‘under eighteen’: *“When I was (…) [daughter’s age] we had jobs (…) whereas life isn’t like that now*, *and I think that has to be acknowledged really within the organisation” (S11)*.

#### Children’s health

Three participants described how having children with health or developmental problems had been particularly difficult during the pandemic. For example, a participant home-schooling a child with autism described how *“he still doesn’t speak so it’s super challenging (…) he broke half of our house*, *all the TVs and iPads and everything (…) because he was so frustrated at home (…) it was challenging emotionally and financially as well” (S5)*.

Another participant, whose child was at a UK boarding school, described how *“the thing that I have been most stressed by is my son’s mental health*. *So I’m thousands of miles away from him*, *I can’t get back to him quickly if he’s poorly*, *and I know that he is really suffering*, *so I’m permanently living with that worry and anxiety in the background” (S3)*. Another described how their child had needed surgery during the pandemic, explaining that *“the medical policy is that (…) sort of secondary care*, *you go back to the UK for (…) we were like*, *okay*, *but there’s a pandemic (…) we can’t go back to the UK ’cause there’s a pandemic (…) I had to fly my [children] and I wasn’t allowed to bring [partner] either*, *they wouldn’t pay for [him] to come back” (S9)*.

### Impacts of the pandemic

#### Financial impact

Many described a negative financial impact of the pandemic. For example, one described setting up their own business overseas which had been severely affected–*“my business completely collapsed (…) [I had to] keep paying the rent [for the business] while making absolutely zero money” (S5)*–whilst another had to give up their work in order to minimise their risk of catching COVID-19 so they could fly back and forth to their children in the UK: *“I’ve had to give up work because it’s simply not safe and therefore we’ve got a massive loss of income” (S3)*.

Quarantine, and COVID testing, were also described as being expensive: *“we’re going back next week to the UK*, *and as a family of two*, *it’s costing us £750 in COVID tests (…) I don’t find that an insignificant amount of money” (S8)*. Some participants reported they risked losing their overseas allowances if they were away from post for two months or more: *“If you’ve been away from post for two months*, *they cut your allowances now (…) there are still costs involved maintaining (…)Internet membership here (…) there’s got to be a cut-off point*, *but there isn’t any flexibility for the circumstances in which you might be back in the UK” (S8)*.

#### Psychological impact

Some participants had found the pressure of the pandemic, coupled with perceived insufficient psychological support, difficult to bear and as a result were not planning to go on overseas postings again in future: *“I’m fairly tough but it’s been so extremely hard and I have asked my husband if we can please go back to the UK (…) I’m not prepared to go to another overseas posting in the current circumstances and I don’t think that message has got through to London yet*, *I don’t think that we’ve received the psychological support as families that we might have” (S3)*.

Living on compounds with many other people was particularly stressful, making participants anxious about catching COVID-19 and leaving them feeling unsafe in their own homes: *“Every time I use my front door*, *I have to put on a mask because I’ve got people [living with me] who have gone home and they’ve gone out partying and they’ve gone to bars and clubs (…) so it’s just permanently living with that added stress when normally in the UK you go home*, *you shut your door*, *you feel safe (…) we don’t have that here at post” (S3)*.

Many reported that their partners had coped better with the pandemic than they had, and so they had turned to their partners for support; *“I think I’m on the stable end of things*, *I wouldn’t have accepted this posting [otherwise] (…) but I have had a pretty hard couple of years (…) and he has been remarkable*, *he’s held it all together and that’s been amazing” (S3)*. One participant described how they believed FCDO staff were resilient by nature: *“When [partner] was recruited there was very much a focus (…) [on] being resilient and adapting to overseas environment*, *living overseas*, *’cause I think it does put stresses and strains on [them] so historically I think they have recruited the right people to cope with potential stressors that come in (…) the systems work because they have resilient people by nature*, *they’ve selected” (S4)*.

However, another participant described their partner struggling during the pandemic: *“[Partner] had it much harder (…) I think she’s struggled a lot (…) mainly because she can’t get out*, *she’s working from home*, *not meeting people (…) in the corridors or (…) having an opportunity to have a coffee with someone*. *And she’s really*, *really struggled” (S2)*. This participant described how their partner’s struggles had impacted them, as they felt guilty for not having been able to do enough to support their partner due to being so busy working on the COVID frontlines themselves: *“She becomes very difficult to talk to*, *and gets very emotional (…) normally we get on really well and we have a great relationship and she’ll always listen to me*, *if I’m saying*, *you’re struggling (…) she will take it on board but this time she actually didn’t and I think it’s the one time that I’ve not really been able to be there for her*, *because of COVID*, *because my job’s frontline (…) as well as sort of be a husband to the wife as well as be a father to the kids and I think it was a bit too much for me to take” (S2)*.

Partners in conflict zones described the pandemic as accentuating other potentially traumatic stressors, describing how they were having to cope with not just the pandemic but many other things on top of that: *“the pandemic is a significant issue*, *but it’s not the only issue that people at post are dealing with (…) the pandemic is only one element to your life when you’re living overseas” (S3)*.

#### Living in lockdown

Participants described various challenges of living in ‘lockdown’, particularly in overseas postings. For example, compound life removed participants from their usual support networks. For those not working, this could result in them feeling very isolated during lockdown as they had no one to talk to: *“since I’m on the compound the only spouse not working*, *it was still kind of lonely in a way (…) I think this had a huge impact (…) having work I think helps because then you can concentrate on this (…) but not having this to distract yourself (…) it felt even more isolating (…) because I didn’t have something specific to do” (S7)*.

The fact that lockdown had been going on for so long in some countries was perceived to affect wellbeing: *“I still don’t think people understand the cumulative effect of the prolonged lockdown and whilst in the UK things have got easier in the last six or seven months (…) we’ve actually been in lockdown with curfews from eight o’clock at night (…) the public parks are closed (…) you’re very much reduced to staying within the confines of your own apartment (…) you can put up with that for four*, *five*, *six months*. *But when for a lot of people it’s been going on for nearly two years*, *it does have an impact on your mental wellbeing” (S8)*.

While lockdown experiences appeared to vary from country to country, all participants described it as challenging. There were benefits of being based in major cities during lockdown, such as *“it’s very sophisticated (…) first class medical support infrastructure was there*. *The schools are quite robust (…) the flick to remote working for [partner] was pretty good because you get mega bandwidth” (S4)* but also challenges of being in this type of urban location: *“I suppose conversely very little space*, *very urban (…) I think [lockdown] got harder the longer it went on” (S4)*.

Participants described various strategies for coping with lockdowns and social restrictions, including art, reading, religious activities, puzzles, writing, and telephone or video calls with friends and family; *“some hobbies (…) just trying to pass the time” (S7); “I gave myself projects (…) you have to be super organised and super structured ‘cause otherwise you go mad” (S3)*. Although many praised technology such as Skype, Zoom, FaceTime or WhatsApp for allowing them to connect with their families–*“I use FaceTime*, *I speak to my daughter every day (…) that’s one of the things that I’m really grateful [for] (…) that keeps us in touch” (S6)*–this was not always an appropriate way of keeping in touch with older family members, who were sometimes *“not really keen on this technology” (S7)*. Other ways of coping included trying to focus on the positives; *“look at the positive side of things or whatever*, *and so there’s always something*, *you can be like*, *‘well*, *at least we’re here’ (…) and so that’s good (…) helps with the mental health side” (S10)*.

### Perceptions of organisational response to COVID-19

Participants acknowledged that the pandemic was a difficult, complicated and unprecedented situation, and there appeared to be a general sense that the organisation had done what it could; *“I don’t think they’re doing a bad job considering (…) there’s so many different embassies and all the other offices they’ve got all around the world and they all have the same problem (…) they’re doing what they can” (S6); “I genuinely think the organisation was pretty good*. *I just think it was slightly reactive rather than proactive*, *but (…) every organisation was probably like that” (S4)*.

However, many participants believed the organisational response was slightly slow and/or reactive; *“I think it has tried to respond as different circumstances emerge*, *but I think in terms of the pandemic*, *they’ve really been very slow off the starting blocks” (S8); “I feel for the first six to seven months of the pandemic things just sort of drifted on*, *‘we’ll wait and see (…) how things develop’*, *there didn’t seem to be any crisis planning (…) I think they’ve been playing catch-up ever since” (S8)*.

Two aspects of the organisational response in particular were discussed in detail by the participants: communication and vaccine provision.

#### Perceptions of organisational communication

Some participants were extremely positive about the communication received from the organisation during the pandemic–for example, *“There’s information coming out virtually every day (…) in all respects*, *even down to what aircraft are flying in from the UK (…) I can’t fault the FCDO (…) they have a hell of a lot of really experienced and some really smart people work for this organisation” (S6)*. Others felt that while the organisational response as a whole had been good, communication had been slow–in particular, certain teams were perceived to have been overwhelmed, thus making them slow to respond: *“it was a bit slow and I think some of the teams became under pressure*. *So as a spouse with a kid in the UK at boarding school and a kid here in private education (…) it was very hard to get responses from the education team because they were probably inundated by thousands*, *thousands of requests for people to sort out the boarding school stuff” (S4)*.

Others felt various aspects of communication could have been improved. For example, one participant felt that the organisation should avoid communications that were too UK-specific: *“In some of the messages that I’ve seen*, *it’s far too London-centric and how busy we are in London and how we’re dealing with this and isn’t it great now we don’t have to wear masks*, *isn’t it great that we’re out of lockdown*? *These messages don’t go down very well with people at post who are facing nightly curfews*, *have got nowhere to go*, *and confined to their apartments” (S8)*. One participant felt they were not kept properly informed about important issues; *“regular updates would actually help (…) out of sight out of mind is how a lot of people actually feel” (S8)*. Another participant felt they needed more communication regarding travel information and requirements: *“there is no formal*, *‘oh we know that you’re a Foreign Office family and therefore we’re going to help you because we know you have to fly back’ (…) there’s nothing*, *there’s no sort of formal communication of information that way” (S3)*. One participant suggested that better communication, demonstrating a better understanding of the issues faced by those at overseas posts, would be helpful: *“I think [it] would be better (…) if they actually (…) were to circulate (…) a summary of what different people across different posts are actually experiencing*, *what are the issues*, *what are the challenges facing people in the overseas network (…) not only that*, *but to say what action points*, *if any*, *from these discussion forums*, *are they going to take forward” (S8)*.

Finally, there appeared to be a general sense that the FCDO frequently relied on employees to relay information to their partners, rather than ensuring themselves that partners were kept up-to-date: *“some [staff] spouses travel a lot and (…) don’t keep abreast of everything that’s going on*. *So I think [partners] can feel slightly in the dark about what’s actually being done in the network (…) I think the Office relies too much on the officer to cascade the information down to their partners” (S8)*.

#### Provision of vaccines

One participant described how getting vaccines out to families at overseas posts had *“not been handled brilliantly” (S3)*, adding that they received their booster three months later than they should have, *“just because they were disorganised because they didn’t think in advance*, *how could we get boosters out to posts in time (…) that’s added unnecessary tension and stress at post because there is no medical care here (…) for us to be months behind when we have no medical care makes me really sad” (S3)*.

Another overseas participant described feeling that booster jabs had been forgotten: *“We got our flu jabs organised by the (…) embassy*, *and the first two COVID injections*, *but then there was no direct guidance as to the booster and I was left sort of scrambling around speaking to other people and we ended up making our own arrangements*, *but again I felt we were just dropped (…) why did we get the first two*, *and then there was absolutely no effort to give us the booster*?*” (S11)*.

#### Availability and perceptions of organisational support

Participants described various avenues of support available to them via the FCDO. One partner praised a confidential helpline which had been set up, saying *“if you feel your mental health is suffering and you want to talk with somebody about it*, *that’s very good” (S8)*. Meetings with the Permanent Under-Secretary (PUS) were also described as helpful, although these were not as frequent as some participants would have liked: *“I think a lot of what they did was a good start*, *so at the beginning of the pandemic*, *the PUS had a meeting with all spouses and partners were invited online*. *And it was understanding*, *it was listening*, *that was very helpful*. *Since then we may have had three other meetings over the space of two years*, *and really given that our lives are so much harder than what we signed up for*, *I really think (…) senior management could have reached out to us more” (S3)*.

Some felt that, as partners of staff rather than staff, it was not always clear what support was available for them from the organisation, particularly those who were not from the UK themselves: *“I know a lot of foreign-born spouses (…) aren’t aware necessarily of what support’s available to them” (S8)*.

Another participant suggested they would have liked more wellbeing events organised by the FCDO; *“For the embassy staff they had often kind of mindfulness (…) sessions*, *but for spouses this wasn’t done*, *so I feel like it would have been nice (…) something like yoga (…) a bit more also for the spouses*, *something kind of wellbeing related” (S7)*. Others simply wanted the organisation to reach out to them more; for example, one participant who had experienced other potentially traumatic circumstances in a hardship posting during the pandemic reported, *“I was surprised that nobody rang me from the Office to say you’ve been through a traumatic experience*, *are you okay*? *I didn’t receive a single phone call from London*, *not one (…) they don’t have any sort of psychological safety net” (S3)*.

#### Barriers to organisational help-seeking

Several participants reported that at their lowest points during the pandemic, they were unable to reach out for help: *“I’m so blessed to have a stable marriage*. *If I didn’t have that and I was alone at post a spouse*, *I’m not sure that I would be able to ring the [Employee Assistance Programme; EAP] and say I need help*, *‘cause that means you have to be the one to motivate yourself and I don’t think I would have the ability to call for help” (S3); “Back then*, *I think it was in a state (…) I wasn’t really sure what to do and who to turn to (…) I just didn’t have the (…) strength to try and call different people and then ask” (S7)*. One participant suggested that people with substantial psychological issues would be unlikely to reach out for help and believed that proactive support would have helped: *“Most people who are in the situation where they’re not feeling well*, *I think they wouldn’t be the ones who are reaching out (…) it would be much easier for them if there is somebody who is trying to contact them” (S7)*.

For non-UK based participants, it was often challenging to obtain support when they tried to. For example, one participant described how *“I spoke to the EAP but they said I would need professional help and so they couldn’t help me*, *and because I’m not in the country*, *I’m not in the UK*, *they couldn’t refer me on (…) you were just left to sort it out yourself really” (S7)*. This participant suggested that it would be useful if there were clear signposting pathways for the overseas network: *“A lot of people (…) don’t really know who to turn to and it’s all well to have something online where people can phone*, *that’s fine*, *but for some (…) they need professional help and there’s (…) this gap so if they can’t refer you on then you’re feeling kind of lost (…) some kind of mental health support that’s available for everybody in their country or at least knowing where to signpost you to (…) so at least also that you don’t have to contact different organisations until you find somebody who can help you (…) something that just kind of*, *okay*, *call this number and then they can sort you out” (S7)*.

### Informal support and help-seeking

#### Diplomatic service families association

The Diplomatic Service Families Association (DSFA) was typically praised for having enabled the development of a community of partners/spouses who could support each other. One participant described how, *“the [DSFA] has been quite extraordinary (…) just one or two colleagues there who have really reached out to spouses and partners who have been struggling and without the DSFA doing that*, *I think a lot of partners really*, *really would have found life extremely difficult (…) we built up a bit of a community of spouses who are struggling and are able to share and sympathise and encourage” (S3)*.

Regular online catch-ups organised by the DSFA were praised as being a *“safe space” (S3)* and *“really helpful and supportive” (S10)*. However, different time-zones could make involvement with the DSFA challenging: *“I used [DSFA support] more when I was in the UK because obviously the time was more friendly*, *like now I think a lot of their events are sort of two in the morning my time” (S10)*.

#### Other informal support networks

Participants described various avenues for seeking informal support, such as others living in their embassies; *“There is official FCDO support*, *and there’s the unofficial*, *you know*, *just people living together and looking after each other” (S6)*. Most also reported accessing support from their partners when they needed it: *“I think [partner] tried to be supportive in a way to kind of lift me up*, *helped me to be more positive” (S7); “[Spouse] is enormously resilient*, *he is just an extremely stable character*, *he always has been and I am very grateful for that (…) he’s been giving me a huge amount of support” (S3)*. For some, this kind of informal support was enough: *“There’s lots and lots of emails about support groups and stuff but (…) I don’t feel as if I need to unburden myself to anyone (…) it’s a nice place to be and I have a few friends that I’ve made out here and we’re all in the same boat so I don’t feel as if I really need any outside help” (S6)*.

However, one participant described difficulties developing their own support network at their partner’s overseas post due to the language barrier; *“It’s still been difficult because (…) most parents [are Southern Europeans] and my [Southern European language] is unfortunately still not great (…) because of the pandemic most language classes are online*, *which I tried but just doesn’t work for me (…) I can read and I’m getting better at understanding*, *but it’s just so hard for me to speak*, *it’s another skill again*, *and because of the pandemic*, *it wasn’t just possible to meet up in a way to learn the language (…) this also made it difficult*, *being in a country where you’re not quite so good in the language and therefore can’t really integrate in their society” (S7)*.

Many had developed their own ‘spouse networks’ which were deemed to be extremely helpful: *“I think between us we’ve all tried to keep an eye on one another (…) and if we haven’t been able to meet at least use WhatsApp or pick up the phone and talk to people (…) just trying to look after each other and being alert to people’s needs*. *I think there has been a strong sense of looking after one another” (S8)*. A WhatsApp group set up specifically for boarding school families was also praised, being referred to as *“a really good resource (…) we’re able to share support and information with one another (…) that’s been a brilliant support*, *just because sharing of information for those who try to travel has been invaluable*, *’cause we feel like we’re scrabbling around for the latest information all the time” (S3)*.

### Suggestions for the future

#### Learning lessons from the pandemic

Several participants emphasised the importance of learning from the experience of COVID-19 to better understand how to cope with future crises. One participant suggested that learning lessons may be difficult given the turnover of staff in the organisation: *“I’d like to think [the FCDO has learned lessons from the pandemic] but unfortunately the nature of the beast in the Foreign Office is like every two*, *every three years people move on and they change jobs and that collective memory disappears very quickly (…) the lessons learned I feel will be lost (…) the intention is there*, *but the reality isn’t and a lot of times you do forget what happened*, *people have got short term memories and it feels as though you’re starting from scratch all over again” (S8)*. Suggestions for better being able to learn lessons included, *“try and call back from the network when (…) a new crisis emerges*, *put out to the network*, *‘look*, *who was involved with COVID*, *would you be willing to be part of a response team in addition [or] instead of your day job*, *to be part of a wider team that deals with an issue that replicates something that’s happened in the past*?*” (S8)*.

#### Understanding the importance of flexibility

Overall, much of the criticism of the organisational response to the pandemic appeared to stem from the perception that various individual, unique needs specific to certain families had not been met. Often, this appeared to be because particular circumstances left participants unable to receive the (practical, rather than psychological) help they needed due to them falling through gaps and not meeting the exact criteria they would need to meet in order to receive the help they needed. For example, the participant whose daughter was over eighteen and thus not classed as a dependant despite very much still depending on her parents, or the participant who could not access the medical treatment their child needed due to their child not being at post with them. Indeed, flexibility appeared to be central to many participants’ suggestions for what the organisation should consider doing in the future–i.e., understanding the varied and unique circumstances faced by many diplomatic families and ensuring no one’s needs were left unmet. For example, the participant who was unable to fly their child out to post due to their child being over eighteen and therefore not counting as a dependant suggested they wanted *“just more support with our circumstances (…) the acknowledgement*, *we do fall through this loophole and (…) someone saying*, *‘right*, *we’ll address that’*, *because I just fear if it happens again it would be the same scenario” (S11)*.

However, many participants acknowledged that the organisation had been flexible during the pandemic, but urged the organisation to consider extending these, due to the pandemic continuing to be an ongoing extraordinary situation and therefore employees and their families still had different needs to their typical, non-pandemic needs. For example, one participant suggested that the organisation should continue being flexible with regards to carrying over leave; *“There was an issue about carrying over leave (…) they have extended up to twenty days a year [you can carry over] which will need to be reviewed (…) the flexibility has been appreciated but I think it’s something they’re talking about*, *it should come to an end at the end of this year [2021] but I think they need to look at it more sympathetically*, *potentially some officers may need to carry forward more than ten days in 2022 or 2023” (S8)*. All participants described the benefits of their partners being able to work from home and felt this flexibility should remain even after the pandemic. It was also felt that the organisation should continue being flexible around the ability to short-tour: *“[The organisation] has agreed recently that you can short-tour if things become operationally difficult for you (…) without incurring any additional costs for so doing*, *whereas in the past*, *if you short-toured (…) there’d be an operational cost to the officer for that (…) they have been good*, *but I think that’s up for review and I think the Office do need to think very carefully about renewing this option to short-tour at no operational costs to the officer” (S8)*. One participant pointed out that *“a lot (…) of the stress the pandemic has brought is due to travel disruption and other practical considerations (…) being separated from partners (…) if the practical issues can be solved*, *the effects on mental health would be greatly reduced” (S10)*.

## Discussion

This study aimed to explore the lived experiences of the partners of FCDO staff during the COVID-19 pandemic, thereby furthering understanding of how to best support diplomatic families during any future prolonged crisis. Our interviews with eleven partners of diplomatic personnel highlighted a number of challenges relating to family circumstances, living situations and COVID-19 restrictions, particularly travel-related restrictions as well as the recognition by participants that the FCDO had made significant efforts to support them in highly challenging circumstances. Due to our broad inclusion criteria, participants had lived in a number of different countries during the pandemic–which we note would have had differing guidance, levels of restrictions and COVID-19 policies. We did note some findings unique to participants’ specific locations, such as the additional stressors described by participants in conflict zones and the extra travel-related challenges facing those trying to travel into COVID-free countries. However, despite the variations in COVID policies, we found that all participants described the pandemic as challenging in a multitude of ways, with a number of similarities across the data regardless of where participants were based.

Participants described a number of travel-related challenges during the pandemic, including strict travel requirements (e.g. paperwork and COVID-19 tests) and rapidly changing travel requirements which differed from country to country; needing to isolate before travel to avoid catching COVID-19; perceived lack of exemptions for diplomatic families; isolation and stress caused by needing to quarantine after travelling; being unable to enter certain countries; and the emotional toll of forced evacuations. Many of these findings are in line with those of other recent studies: for example, border closures have been found to be associated with high levels of distress and low wellbeing among people wanting to enter or leave Australia [37] and being stranded abroad during the pandemic appears to be associated with high levels of depression, anxiety and stress in citizens stranded for reasons such as tourism, business travel, studying abroad or long-term employment abroad [38]. Our participants emphasised how travel restrictions, and other deployment-related challenges, resulted in feelings of powerlessness and not being in control of their own lives.

The rationale behind ‘living with COVID-19’ and relaxing many social restrictions while also keeping strict travel regulations in place, was queried. This created understandable frustration, especially given the mixed evidence on how effective travel restrictions are in containing infections, and growing evidence of their negative impact on economies and diplomatic relationships [39].

Quarantine was described to be isolating, lonely, tedious, stressful and frustrating–this is unsurprising given the evidence of the psychological impact that quarantine can have [40], particularly for individuals in quarantine outside of their home countries [41]. Participants reported distress at needing to quarantine in hotels if they had empty accommodation waiting for them, and some were disappointed the organisation would only pay for UK quarantine.

Forced evacuation of families was described as especially upsetting, especially during evacuations from countries with relatively few cases of COVID-19 when the UK was perceived as being more dangerous. We acknowledge these perceptions do not take into account some complex issues which the FCDO would have been grappling with, such as the ability to Medevac, failings in country health systems and duty of care requirements. Previous literature has discussed the potential distress involved during abrupt repatriations, including studies on migrant workers [42] and college students who had been studying abroad [26]. Our findings support the idea that experiencing such a sudden change during an already uncertain and stressful time can be detrimental to individuals’ psychological wellbeing.

Numerous stressors relating to family life, childcare, schooling, and family healthcare were described, and the impact of these should not be overlooked; it has been suggested that individuals managing a greater number of challenges during the pandemic–including periods of separation from loved ones, poor support and caring for children with additional needs–are likely to be particularly vulnerable to experiencing negative psychological effects [43]. We identified that participants with children in UK boarding schools felt more could have been done to fly their families out to them. Home-schooling was also described as difficult due to juggling the need for home-schooling with work and other family responsibilities. This is very much in keeping with previous research which found that parents involved in home-schooling during the COVID-19 pandemic experienced higher levels of distress than those not home-schooling or those without children [44]. Others reported the financial burden of flying back and forth to the UK every school holiday in order to care for boarding school children. Our finding that partners took on more childcare responsibilities due to their diplomatic spouses having increased workloads echoed findings from partners of other frontline workers during the pandemic: for example, partners of healthcare workers have reported that their partners’ increased workload meant that they had to take on more of the domestic responsibilities that were previously shared [45, 46].

Participants described negative financial impacts of the pandemic, citing not only the costs associated with travelling, quarantine and COVID-19 testing but also losing their employment or having to give up work in order to care for children. Similar concerns have been noted in abruptly repatriated college students [26]. Financial strain and income loss have been associated with high levels of depression, anxiety and distress during the pandemic [47]. Many negative psychological impacts of the pandemic were also described. In particular, participants located in hardship postings coping with other potentially traumatic stressors and those living on busy compounds who feared exposure to COVID-19 appeared to struggle. Many participants reported feeling isolated and frustrated during lockdown; ways of coping included keeping in touch with loved ones, keeping busy, and positive thinking. These align with the key coping strategies described by repatriated college students: seeking social support, keeping busy and positive reframing of the situation [26].

Overall, many participants acknowledged that senior management of the FCDO had been in a difficult position during the pandemic and many felt they had coped as well as could be expected. However, issues were raised with the speed and transparency of communication from the organisation and lack of clear guidance around vaccines. This is perhaps unsurprising as the pandemic and associated social restrictions are unprecedented and organisations are unlikely to have been prepared for a public health emergency of such magnitude. Indeed, research suggests other organisations, such as healthcare organisations, have also been slow to communicate with their staff about policies and risks [48].

Participants described mixed feelings around organisational support; some aspects of this were perceived as helpful (e.g. the helpline), other aspects were seen as helpful but not frequent enough (meetings with the head of service); and some participants described feeling unclear about what was available to them and felt they had not been reached out to. The DSFA was perceived as helpful, although different time-zones hindered some being able to regularly take advantage of their meetings. Mostly, participants appeared to rely on informal support from their partners and informal ‘spouse networks’: we note that maintaining communication with support networks has also been seen as crucial for military spouses during the pandemic [25]. Indeed, social support could be an important resource for diplomatic families to draw upon during a prolonged crisis. Research on international business travellers in terrorism-endangered countries suggests that social networks are particularly beneficial during times of threat and uncertainty [49]. Sense of community and connectedness within the community can be helpful and create a ‘surrogate family’ for spouses [34, 50]; however, building these connections can be difficult for partners who are introverted or reserved in personality [50] and would have been difficult for everyone during COVID-19 when social interactions were so restricted. Our study also revealed that those who had recently moved to new postings, especially those unable to speak the local language reported particular difficulties accessing informal social support within their communities.

Finally, participants discussed their thoughts on the future and how their organisation could better support them if another prolonged crisis were to occur. They emphasised the importance of learning lessons from the COVID-19 pandemic in order to ensure the organisation is better prepared for any similar prolonged crisis that may occur in the future. What appeared to be particularly important was organisational flexibility and understanding about the many different and varied circumstances experienced by diplomatic families during the pandemic, and making sure all are equally supported, with nobody ‘slipping through the cracks’ in terms of either the practical or psychological support needed. Many hoped the organisation would consider extending the flexibilities given to their officer partners during the pandemic, regarding carrying over leave; the ability to short-tour; and working from home. Indeed, diplomatic personnel themselves have reported wanting their employers to continue allowing them flexibility to work from home if desired [51].

Many of the challenges reported by our participants can be related back to extant literature on cross-cultural transitions. For example, we found that evacuation, quarantine, travel restrictions and living in ‘lockdown’–all of which are likely to have substantially impacted the ability of spouses to engage with familiar support systems and access resources–appeared to intensify feelings of loneliness, distress and disconnection. This aligns with previous literature on diplomatic and military spouses’ cross-cultural transitions, which has highlighted the importance of support networks in facilitating adjustment and wellbeing [4, 14, 16–18]. We found evidence that participants struggled to find support during the pandemic; existing literature suggests that even outside of the context of a global pandemic, it can be difficult to establish support networks when frequently moving from country to country [12]. Indeed, the absence of such formal support appeared to compound the challenges faced by spouses during the pandemic. Several of our participants had found informal support networks extremely valuable, highlighting the importance of social support in facilitating coping and adjustment [4].

### Theoretical and practical implications

Based on our findings, we developed a list of recommendations for supporting employees during a prolonged crisis. While our findings were specifically from spouses in diplomatic organisations, these recommendations are also likely to be applicable to other organisations which offer opportunities for individuals and their families to relocate abroad.

Firstly, it would be useful during any prolonged crisis impacting travel for diplomatic (and similar) organisations to ensure staff and their families are kept clearly informed about what is being done to improve staff, and family members’, ability to travel in the face of ongoing restrictions.

It would be helpful for organisations to provide advice to quarantining families about how they can best cope with quarantine–coping strategies might include trying to maintain routines, using the time to develop new skills, taking exercise, reaching out to friends and family via phone calls and video calls, and activities such as reading, writing and puzzles to keep busy [52]. While this suggestion is particularly pertinent to diplomatic and similar organisations, whose families are away from their usual support networks, the advice is relevant to all individuals required to self-isolate [40].

We suggest that the rationale behind evacuation decisions should be clearly communicated with staff and their families. Partners in our study who were evacuated despite wanting to stay at post were left feeling they had no control over their own lives, and also experienced disruption to their employment due to evacuation. Being away from familial support networks can be difficult at the best of times, but particularly during times of crisis or heightened need, and family support can indeed be an essential source of support during times of great stress [52]. Therefore, evacuation should be a last possible resort and should be discussed at every step with the families involved. Where evacuation is necessary, organisations should ensure that staff and their families have appropriate support systems in place. When separation of families is unavoidable, both employees and partners should be supported by the organisation, who could help families to manage separations by encouraging them to develop new routines; ensuring the deployed person is involved in what is going on at home as much as possible; ensuring both partners have information about support; and encouraging them to talk to others in similar situations [43]. It has been suggested that expertise from the military could help other separated families during the pandemic [43] and so diplomatic and similar organisations could consider liaising with military organisations for advice and consider adapting family resilience programmes developed by the military.

Our findings highlighted a number of ways in which the organisational response to the pandemic, including the support provided to families, was perceived as lacking. We recommend that diplomatic and similar organisations are cognisant of the many different circumstances their employees’ families are in and ensure that appropriate support is in place for all of them. Good communication with staff and family members about these issues is likely to be helpful even if organisations cannot necessarily provide the solutions that are desired. Implementing policies to support the basic needs of expatriate families would help to demonstrate commitment to supporting the family unit. Organisations should provide appropriate psychological support / signposting to professional support services; increase resources to help staff and their families; and ensure that all forms of support are advertised throughout the network, perhaps in circular emails, newsletters or on the organisational intranet. It would also be helpful to provide families with lists of resources and useful contacts and clarity around what is provided by the different services available.

We acknowledge there is little that can be done to remove the negative financial impact of the pandemic. However, diplomatic and similar organisations could consider subsidising some of the expenses participants had (e.g. COVID-19 tests, quarantine, temporary accommodation, childcare) or lobbying for more exemptions for employees’ families which might reduce the need for expensive testing and quarantining.

We note that good communication is essential during a stressful, evolving crisis situation and has been recommended as a crucial tool for supporting the wellbeing of (military) families [43, 53]. We recommend that organisations review their COVID-19 experience, analyse what went well and what could have been done better, and learn lessons from the pandemic to ensure that they are well-equipped to respond to any prolonged crisis which might occur in the future; indeed, ensuring that organisational lessons are learned was also one of the key suggestions made by participants themselves. Effective, proactive, regular communication during a crisis appears to be especially important.

We recommend that the FCDO and similar organisations consider how to facilitate informal support networks, hosting remote introductory events and ensuring families are connected (remotely) with other local families or families in similar circumstances. It could be useful to facilitate the setting up of informal support networks (such as WhatsApp groups) for specific groups of people–for example, groups specifically for those experiencing separation from families, or those with young children, older children, or children with special educational needs. These should be well advertised to ensure they are accessible to all who might need them. These could provide readily remotely available resources for support and advice during any crisis. This is important as social support from people facing similar challenges is likely to be helpful [54]. The evidence that such groups can be helpful comes from our participants themselves, several of whom spoke very highly of an informal WhatsApp group set up specifically for boarding school parents. Diplomatic and similar organisations could also provide specific training for staff and spouses regarding how to develop new, socially supportive group memberships (or maintain existing ones) when deployed to new posts. Interventions designed to increase social group membership can be protective against changes brought about by major life events: for example, the ‘Groups 4 Health’ intervention targeting group-based belonging has been found to protect against loneliness and depression during the COVID-19 pandemic [55, 56].

### Theoretical and practical contributions

To our knowledge, this is the first study to explore the lived experiences of diplomatic families during a prolonged crisis, therefore filling a gap in the literature by establishing the specific needs of partners of diplomatic personnel during and after a prolonged crisis such as the COVID-19 pandemic. Much of the existing research fails to consider the experiences of expatriate spouses, focusing instead on the employed partners. For example, research on military families tends to focus on the service members themselves; when spouses and families are discussed, it is often through a service member-focused lens, focusing on how spouses are affected by the absence of their deployed partner or how the service members’ health impacts on that of the spouse [23]. This study provided a voice to the often overlooked romantic partners and is therefore an important addition to literature on cross-cultural transitions, both generally and within the context of a prolonged crisis.

The semi-structured design of the interviews allowed for great flexibility in the data collection, giving participants themselves the ability to direct the flow of the interview and ensure that they covered the topics most important to them.

Good rapport with participants was built and continued throughout the study, beginning with informal emails to confirm eligibility and arrange interview times, and continuing throughout the interviews. During the interviews, the interviewer was friendly, non-judgmental and interested in what the participants had to say. This helped the participants to feel comfortable sharing their personal stories and allowed for the collection of rich data.

### Limitations

There are several limitations to this study: first, transcripts were independently coded by only one author. Ideally, a double-coding process would help to minimise potential bias in coding. However, the second and third authors read through the list of themes and quotes chosen to reflect each theme and were in agreement that they made sense.

The sample size was small (n = 11), but qualitative studies can benefit from having small population sizes as it is easier for the interviewer to develop rapport with all participants [57]. Despite the small number of participants, the researchers were satisfied that no new themes were emerging from the data and they had reached data saturation by the end of the 11 interviews [58]. However, we acknowledge that there is debate about what ‘data saturation’ means and that the way in which researchers conceptualise their themes can affect how early ‘saturation’ is identified [59].

We also note that our 11 participants may not necessarily have been representative of FCDO partners in general: selection bias is a possibility, in that those with particularly strong views may have been more likely to volunteer. For example, it is possible that individuals who faced particularly extreme challenges may have been more likely to volunteer, due to their experiences making their views especially strong. Such individuals may also have been more likely to volunteer than those who faced fewer challenges due to perceptions of what the researchers wanted to hear: those with greater challenges may have been confident they had plenty of things to say in their interviews, while others who faced fewer challenges may have thought they did not have much to say and may have assumed the researchers would be less interested in their stories. Additionally, those with stronger opinions on FCDO culture, support, and COVID-19 impacts may have been more likely to volunteer, while those with less extreme opinions may not have volunteered. Additionally, barriers to support may well be more severe in the wider population of spouses: individuals who are uncomfortable discussing mental health issues (and therefore likely to have avoided seeking psychological support) are unlikely to have volunteered to participate. We also note that data from a larger study population may have yielded more information on the similarities and differences between experiences in different locations. This would have allowed us to better understand how specific location might relate to wellbeing during a time of prolonged crisis.

In accordance with ethical guidelines, participants were assured of confidentiality and anonymity throughout the study and in the write-up: however, some may have remained concerned that they may be able to be identified through their quotes, and so may have held back information that they felt was controversial. Participants may also have mitigated their responses according to what they imagined the interviewer wanted to hear. It is therefore possible that certain issues may have been downplayed or omitted from participants’ accounts altogether due to concerns about confidentiality or social desirability bias.

## Conclusions

Our interviews with 11 partners of diplomatic personnel revealed a number of challenges faced by diplomatic families during the COVID-19 pandemic. Our findings suggest that diplomatic (and similar) organisations can best support their staff and their families during a prolonged crisis by: keeping families clearly informed about travel restrictions and requirements; providing practical advice about coping with quarantine; support during evacuations/separation from family, including encouragement to talk to others in similar situations; understanding and good communication regarding potential family-related challenges such as childcare; subsiding crisis-related expenses where possible; good communication around policies; reviewing their experience during the crisis to ensure lessons are learned; facilitating informal support networks to ensure families are connected with others in similar circumstances; and being flexible and taking individual circumstances into account where possible. The findings are still relevant and important beyond the context of a prolonged crisis, highlighting the importance of organisational support and informal support networks for expatriate spouses to support their psychological wellbeing. Many of our recommendations–such as keeping families informed about policy changes and updates, providing the rationale behind decisions, ensuring families know what support is available, good clear communication, and facilitating informal support networks–are not limited to the pandemic context. Rather, diplomatic and similar organisations can learn from the pandemic experience and use these lessons to develop strategies to ensure that expatriate families are well-equipped to adjust and adapt to new countries and new situations.

## Supporting information

S1 File Clinical studies checklist. (DOCX)

S2 File STROBE checklist. (DOCX)

## Abbreviations

COVID-19 / SARS-CoV-2: novel coronavirus emerging in 2019
DSFA: Diplomatic Service Families Association
FCDO: Foreign, Commonwealth and Development Office
UK: United Kingdom
