# Supplementary material for: “Exceptionally challenging time for all of us”: Qualitative study of the COVID-19 experiences of partners of diplomatic personnel
Source: PLoS One. 2023 Nov 2;18(11):e0293557. doi: 10.1371/journal.pone.0293557 (PMC10621840; doi:10.1371/journal.pone.0293557)
Supplement: S2 File — (DOCX) [file pone.0293557.s002.docx]

STROBE Statement—checklist of items that should be included in reports of observational studies

|  | Item No. | Recommendation | Page  No. | Relevant text from manuscript |
| --- | --- | --- | --- | --- |
| **Title and abstract** | 1 | (*a*) Indicate the study’s design with a commonly used term in the title or the abstract | 1 | Title: “Qualitative study of…” |
|  |  | (*b*) Provide in the abstract an informative and balanced summary of what was done and what was found | 2 | Entire abstract |
| Introduction | | | |  |
| Background/rationale | 2 | Explain the scientific background and rationale for the investigation being reported | 3-5 | Scientific background: Citations throughout Introduction  Rationale: “To date, relatively little is known about the COVID-19 experiences of partners of international travellers. To our knowledge, there is no published academic literature exploring the impact of COVID-19 on diplomatic spouses; again, we can turn to military literature given the potential similarities between diplomatic spouses and those of Armed Forces personnel. Research suggests the pandemic has intensified many of the pre-existing concerns common within military families – such as military spouse unemployment/under-employment, time away from families, child-care challenges and financial concerns [19].” |
| Objectives | 3 | State specific objectives, including any prespecified hypotheses | 5 | Objectives: “The overall aim of this study was to use these findings to develop recommendations for how the FCDO – and similar organisations – could best support their staff during a prolonged crisis such as a pandemic”  Hypothesis: Not appropriate for qualitative study |
| Methods | | | |  |
| Study design | 4 | Present key elements of study design early in the paper | 5 | “This study collected qualitative data using semi-structured interviews” |
| Setting | 5 | Describe the setting, locations, and relevant dates, including periods of recruitment, exposure, follow-up, and data collection | 6 | “Eligible people were aged 18 or over and had a partner employed by the Foreign, Commonwealth and Development Office (FCDO) both currently and for at least six months”  “In September 2021, welfare staff at the FCDO emailed the invitation to 100 randomly selected diplomatic staff who met the inclusion criteria and due to low response rate, two further rounds of invitations were sent in November 2021 and January 2022. The Diplomatic Service Families Association (DSFA) newsletter also published the invitation during this time period”  “Interviews were carried out by the first author between September 2021-February 2022. Most (n=10) took place over Microsoft Teams whilst one took place over Zoom due to the participant lacking access to Teams” |
| Participants | 6 | (*a*) *Cohort study*—Give the eligibility criteria, and the sources and methods of selection of participants. Describe methods of follow-up  *Case-control study*—Give the eligibility criteria, and the sources and methods of case ascertainment and control selection. Give the rationale for the choice of cases and controls  *Cross-sectional study*—Give the eligibility criteria, and the sources and methods of selection of participants | 6 | “Eligible people were aged 18 or over and had a partner employed by the Foreign, Commonwealth and Development Office (FCDO) both currently and for at least six months. In order to recruit participants with a broad range of experiences, there were no inclusion criteria relating to whether participants had been abroad or in the United Kingdom (UK) during the pandemic, the countries they were based in, or the grades/roles of their partners within the FCDO”  “An invitation letter was created by the authors which summarised the aims and proposed methodology of the study and provided the researchers’ contact details, asking anyone interested in taking part to contact the researchers of their own volition. As we were recruiting FCDO staff themselves for a similar study at the same time, the invitation letters explained that we were interested in recruiting both staff and their partners and invited any staff who received the invite to share it with their partners.” |
|  |  | (*b*) *Cohort study*—For matched studies, give matching criteria and number of exposed and unexposed  *Case-control study*—For matched studies, give matching criteria and the number of controls per case | N/A |  |
| Variables | 7 | Clearly define all outcomes, exposures, predictors, potential confounders, and effect modifiers. Give diagnostic criteria, if applicable | N/A | Qualitative research – not applicable |
| Data sources/ measurement | 8* | For each variable of interest, give sources of data and details of methods of assessment (measurement). Describe comparability of assessment methods if there is more than one group | 7 | Data is the transcripts: “All interviews were recorded and transcribed verbatim by the first author with identifying information removed from the transcripts and replaced with ‘[redacted]’” |
| Bias | 9 | Describe any efforts to address potential sources of bias | 7-8 | “Immediately after each interview, the research team created memos in NVivo to record observations of the interviews: these included the most salient issues arising from the interview, initial thoughts about potential themes arising from the data, and overall thoughts on the interview. This allowed the research team to reflect on their interview technique; consider whether any questions should be added, removed or improved; and reflect on how their own experiences or expectations may have influenced either their interactions with participants or their interpretation of the data” |
| Study size | 10 | Explain how the study size was arrived at | 6 | “Recruitment ended when data saturation was considered to have been achieved, i.e. no new information was emerging from interviews.” |

Continued on next page

| Quantitative variables | 11 | Explain how quantitative variables were handled in the analyses. If applicable, describe which groupings were chosen and why | N/A |  |
| --- | --- | --- | --- | --- |
| Statistical methods | 12 | (*a*) Describe all statistical methods, including those used to control for confounding | N/A |  |
|  |  | (*b*) Describe any methods used to examine subgroups and interactions | N/A |  |
|  |  | (*c*) Explain how missing data were addressed | N/A |  |
|  |  | (*d*) *Cohort study*—If applicable, explain how loss to follow-up was addressed  *Case-control study*—If applicable, explain how matching of cases and controls was addressed  *Cross-sectional study*—If applicable, describe analytical methods taking account of sampling strategy | N/A |  |
|  |  | (*e*) Describe any sensitivity analyses | N/A |  |
| Results | | | | |
| Participants | 13* | (a) Report numbers of individuals at each stage of study—eg numbers potentially eligible, examined for eligibility, confirmed eligible, included in the study, completing follow-up, and analysed | 8 | “Twenty-one partners of FCDO staff contacted the researchers for further information about the study. As we do not know how many partners saw the DFSA advertisement or how many FCDO staff shared the invites with partners, it is not possible to calculate an overall response rate. Of the 21 who contacted the researchers, eleven (52%) agreed to take part” |
|  |  | (b) Give reasons for non-participation at each stage | N/A |  |
|  |  | (c) Consider use of a flow diagram | N/A | Not considered necessary given the small population, only one time-point of data collection and voluntary participation |
| Descriptive data | 14* | (a) Give characteristics of study participants (eg demographic, clinical, social) and information on exposures and potential confounders | 8 | “five (45.4%) were male and six (54.5%) were female; ages ranged from early 20s to mid-60s (mean 45) and their partners worked in a wide range of roles and grades within the FCDO (no specific details are presented in order to protect the identities of participants). Between them, participants had resided in 14 different countries across six continents during the pandemic (this number is greater than the number of participants, as several had relocated at least once during the pandemic).” |
|  |  | (b) Indicate number of participants with missing data for each variable of interest | N/A |  |
|  |  | (c) *Cohort study*—Summarise follow-up time (eg, average and total amount) | N/A |  |
| Outcome data | 15* | *Cohort study*—Report numbers of outcome events or summary measures over time | N/A |  |
|  |  | *Case-control study—*Report numbers in each exposure category, or summary measures of exposure | N/A |  |
|  |  | *Cross-sectional study—*Report numbers of outcome events or summary measures | N/A |  |
| Main results | 16 | (*a*) Give unadjusted estimates and, if applicable, confounder-adjusted estimates and their precision (eg, 95% confidence interval). Make clear which confounders were adjusted for and why they were included | N/A |  |
|  |  | (*b*) Report category boundaries when continuous variables were categorized | N/A |  |
|  |  | (*c*) If relevant, consider translating estimates of relative risk into absolute risk for a meaningful time period | N/A |  |

Continued on next page

| Other analyses | 17 | Report other analyses done—eg analyses of subgroups and interactions, and sensitivity analyses | N/A |  |
| --- | --- | --- | --- | --- |
| Discussion | | | | |
| Key results | 18 | Summarise key results with reference to study objectives | 27 | “This study aimed to explore the lived experiences of the partners of FCDO staff during the COVID-19 pandemic, thereby furthering understanding of how to best support diplomatic families during any future prolonged crisis. Our interviews with eleven partners of diplomatic personnel highlighted a number of challenges relating to family circumstances, living situations and COVID-19 restrictions, particularly travel-related restrictions as well as the recognition by participants that the FCDO had made significant efforts to support them in highly challenging circumstances” |
| Limitations | 19 | Discuss limitations of the study, taking into account sources of potential bias or imprecision. Discuss both direction and magnitude of any potential bias | 33 | Entire ‘Limitations’ subsection |
| Interpretation | 20 | Give a cautious overall interpretation of results considering objectives, limitations, multiplicity of analyses, results from similar studies, and other relevant evidence | 33-34 | Entire ‘Implications’ subsection |
| Generalisability | 21 | Discuss the generalisability (external validity) of the study results | N/A |  |
| Other information | |  | | |
| Funding | 22 | Give the source of funding and the role of the funders for the present study and, if applicable, for the original study on which the present article is based | Application | Funding and role of funders is described in the application. |

*Give information separately for cases and controls in case-control studies and, if applicable, for exposed and unexposed groups in cohort and cross-sectional studies.

**Note:** An Explanation and Elaboration article discusses each checklist item and gives methodological background and published examples of transparent reporting. The STROBE checklist is best used in conjunction with this article (freely available on the Web sites of PLoS Medicine at http://www.plosmedicine.org/, Annals of Internal Medicine at http://www.annals.org/, and Epidemiology at http://www.epidem.com/). Information on the STROBE Initiative is available at www.strobe-statement.org.
